# Genetic correlation and causality assessment between Alzheimer’s disease and asthma

**DOI:** 10.1002/alz.089984

**Published:** 2025-01-03

**Authors:** Olasunkanmi David Bamidele, Emmanuel O Adewuyi, Tenielle Porter, Ayeisha Milligan Armstrong, Giuseppe Verdile, Simon M. Laws, Neurological Conditions Group

**Affiliations:** ^1^ Centre for Precision Health, Edith Cowan University, Joondalup, Western Australia Australia; ^2^ Faculty of Pharmaceutical Sciences, University of Ilorin, Ilorin, Kwara Nigeria; ^3^ Curtin Medical School, Curtin University, Bentley, Western Australia Australia; ^4^ Collaborative Genomics and Translation Group, School of Medical and Health Sciences, Edith Cowan University, Joondalup, Western Australia Australia; ^5^ Curtin Health Innovation Research Institute, Curtin University, Bentley, Western Australia Australia

## Abstract

**Background:**

Observational studies have suggested a co‐occurring relationship between Alzheimer’s disease (AD) and asthma. However, the aetiology and biological mechanisms underlying AD and its potential association with asthma, an autoimmune condition, remain unclear.

**Method:**

We examine the genetic relationship between AD and asthma by analysing large‐scale genome‐wide association study (GWAS) summary data from international research consortia and groups. We conducted a linkage disequilibrium score regression (LDSC) analysis to assess AD’s genome‐wide (global) genetic correlation with asthma. We also performed a two‐sample Mendelian randomization (MR) analysis to investigate the potential bi‐directional causal relationships between AD and asthma. We performed additional analyses for possible (partial) replication of our genetic correlation and causality findings on the side of both AD and asthma.

**Result:**

Our LDSC‐based genetic correlation estimates revealed a positive and significant global genetic correlation (*r*_G_) of AD with asthma (*r*_G_ = 0.19, *SE* = 0.04, P = 5.57 × 10^‐6^). We used additional asthma GWAS data for replication testing, with consistent, significant results across all data. We observed a similar pattern of correlation estimate direction in our (partial) replication testing for AD (clinically diagnosed cases), although only one of the asthma GWAS data’s results was significant (likely due to a smaller sample size). Based on the inverse‐variance weighted (IVW) model, our MR analysis found no evidence for a significant causal association between AD and asthma. This MR result was consistent with AD or asthma as an exposure or outcome variable, respectively. Sensitivity testing using other MR approaches produced results similar to our IVW model, supporting no evidence of a causal association between AD and asthma.

**Conclusion:**

Our study reveals a significant genetic correlation but non‐causal association between AD and asthma, implicating the presence of shared genetic mechanisms and biological pathways in both conditions.

